# Correlation between islet α cell function and peripheral neuropathy in patients with type 2 diabetes mellitus

**DOI:** 10.1016/j.clinsp.2024.100392

**Published:** 2024-06-21

**Authors:** Yurou Cao, Xueqin Wang

**Affiliations:** a Department of Endocrinology, Jiangsu Rudong County People's Hospital, Jiangsu Province, PR China; b Department of Endocrinology, Affiliated Hospital 2 of Nantong University, and First People's Hospital of Nantong, Jiangsu Province, PR China

**Keywords:** Islet α-cell function, Glucagon, Type 2 diabetes, Peripheral neuropathy, Peripheral nerve conduction velocity, Peripheral nerve conduction latency, Peripheral nerve conduction amplitude

## Abstract

**Background:**

This study explored the correlation between pancreatic islet α cell function, as reflected by the plasma glucagon levels, and Diabetic Peripheral Neuropathy (DPN) in patients with Type 2 Diabetes Mellitus (T2DM).

**Methods:**

A total of 358 patients with T2DM were retrospectively enrolled in this study and divided into the Non-DPN (NDPN) group (n = 220) and the DPN group (n = 138). All patients underwent an oral glucose tolerance test to detect levels of blood glucose, insulin and glucagon, and the Area Under the Curve (AUC) for Glucagon (AUCglu) was used to estimate the overall glucagon level. The Peripheral Nerve Conduction Velocity (PNCV), Amplitude (PNCA) and Latency (PNCL) were obtained with electromyography, and their Z scores were calculated.

**Results:**

There were significant differences regarding the age, disease duration, serum levels of alanine aminotransferase, aspartate aminotransferase, urea nitrogen, high-density lipoprotein, and 2h-C peptide between these two groups (p < 0.05). The NDPN group had higher glucagon levels at 30, 60 and 120 min and AUCglu (p < 0.05). The Z-scores of PNCV and PNCA showed an increasing trend (p < 0.05), while the Z-score of PNCL showed a decreasing trend (p < 0.05). The glucagon levels were positively correlated with PNCV and PNCA, but negatively correlated with PNCL, with Gluca30min having the strongest correlation (p < 0.05). Gluca30min was independently related to PNCV, PNCL, PNCA and DPN, respectively (p < 0.05). The function of pancreatic α islet cells, as reflected by the plasma glucagon level, is closely related to the occurrence of DPN in T2DM patients.

**Conclusion:**

Gluca30min may be a potentially valuable independent predictor for the occurrence of DPN.

## Highlights

• The correlation between pancreatic islet α cell function and diabetic peripheral neuropathy
• Gluca30min may be a potentially valuable independent predictor for the occurrence of DPN

## Introduction

Diabetes Mellitus (DM) is a worldwide health concern with an estimated prevalence of 9.3% in 2019, which is projected to increase to 10.3% by 2030.^1^ Three major types of DM have been categorized, type 1 (i.e. juvenile or insulin-dependent DM), type 2 (i.e., adult-onset DM), and gestational DM (i.e. DM with pregnancy), among which type 2 Diabetes Mellitus (T2DM), which is characterized by insulin resistance and beta cell dysfunction, is the most common DM, accounting for 90% of all DM types.^2^ With the progress of the disease, T2DM patients can develop a number of complications including retinopathy, nephropathy, heart attack and stroke, and neuropathy. Among these complications, Diabetic Peripheral Neuropathy (DPN), which is primarily characterized by pain, paresthesia and sensory loss, is the most common microvascular complication, affecting approximately 50% of T2DM patients.^3^ In addition, DPN is the major cause of gait disturbance, fall-related injuries, foot ulcers, and amputation-related disability in T2DM patients.^4^ As a result, DPN is significantly associated with disease-related health cost and limited quality of life of T2DM patients.^5^ DPN is usually asymptomatic during early stages but cannot be reversed once symptoms appear.^6^ Thus, early detection and timely intervention of DPN are of great significance to the prognosis of T2DM patients with DPN.

One major function of pancreatic islet α cells, which accounts for 33%‒46% of islet cells in humans,^7^ is to secrete glucagon. Glucagon is a 29-amino acid linear polypeptide hormone; it participates in the regulation of blood glucose concentration as the counter-regulatory hormone of insulin,^8^ thus playing a key role in maintaining glucose homeostasis. Mechanistically, glucagon mainly promotes hepatic glucose output by increasing gluconeogenesis and glycogenolysis and reducing glycolysis and glycogen synthesis, leading to increased plasma glucose concentrations.^9^ Previous studies have shown that T2DM is commonly accompanied by hyperglucagonemia,^10^^,^^11^ which in turn reduces the sensitivity of T2DM patients to insulin. Therefore, hyperglucagonemia is considered to contribute to the development and progression of T2DM.^11^^,^^12^ While hyperglycemia is well recognized as a risk factor for the development and progression of DPN,^13^ whether the blood level of glucagon is directly linked to the pathogenesis of DPN is not well studied, although previous studies have suggested a close correlation of hyperglucagonemia with the development of hyperglycemia.^2^^,^^10^^,^^12^

Here, the authors investigated the potential correlation between the function of pancreatic islet α cells, as reflected by the plasma glucagon level, and DPN in T2DM patients. The authors found that plasma glucagon levels were significantly correlated with Peripheral Nerve Conduction Velocity (PNCV), Peripheral Nerve Conduction Latency (PNCA), and Peripheral Nerve Conduction Amplitude (PNCL) in T2DM patients with DPN. The present findings suggest that pancreatic isled α-cell function is closely associated with DPN in T2DM patients and that plasma glucagon levels may be a potential valuable predictor for the development of DPN.

## Materials and methods

### Patient selection and grouping

A total of 457 patients hospitalized in the Endocrinology Department of Nantong First People's Hospital between October 2022 and October 2023 were initially selected for this study. Enrollment criteria for patients included age between 18 and 80 years old and having T2DM, which was diagnosed based on the 1999 diagnostic criteria of the World Health Organization (WHO).^14^ Patients who had one or more of the following health issues were excluded from this study: 1) T1DM, secondary diabetes or other types of diabetes, 2) Acute complications of diabetes such as hyperglycemia and hyperosmolar state, diabetic ketoacidosis, 3) History of malignant tumors, 4) History of serious cardiovascular and cerebrovascular diseases, 5) Severe Hepatic and renal insufficiency, 6) Any diseases affecting glucose metabolism such as hyperthyroidism, hypothyroidism, Cushing's syndrome,7) History of use of neurotoxic drugs, 8) Vitamin B12 deficiency, 9) Any systemic disease related to peripheral neuropathy, 10) Any neuromuscular diagnostic diseases, 11) Pregnant and lactating women, 12) Cervical and lumbar spine diseases (i.e., compression, stenosis, or degeneration), 13) Connective tissue diseases, and 14) Poor blood sugar control and use of GLP-1 receptor agonists or DPP4 inhibitors. After the application of inclusion and exclusion criteria, a total of 358 patients were eventually enrolled in this study. The study design was reviewed and approved by the Human Study Review Committee of Affiliated Hospital 2 of Nantong University. The conduction of the study adhered to the Declaration of Helsinki involving research of human subjects. Informed consent was obtained from all participants prior to the initiation of this study.

The enrolled T2DM patients, based on whether having DPN or not as diagnosed with criteria for DPN,^15^ were divided into two groups, the DPN group (n = 138) and the non-DPN (NDPN) group (n = 220).

### Data collection

Demographic and baseline clinical characteristics of all patients, including age, gender, disease duration, blood pressure, Body Mass Index (BMI), diabetes treatment plan, serum levels of Alanine Aminotransferase (ALT), Aspartate Aminotransferase (AST), urea nitrogen, Serum creatinine (Scr), blood Uric Acid (UA), blood lipid profile, glycated Hemoglobin (HbA1C), Peripheral Nerve Conduction Velocity (PNCV), Peripheral Nerve Conduction Amplitude (PNCA), and Peripheral Nerve Conduction Latency (PNCL), were collected from the medical record of each participant.

### Oral glucose tolerance test (OGTT)

All patients underwent an oral glucose tolerance test. Briefly, patients fasted for at least 10 hours overnight and were instructed to drink 250 mL water that contained 75g of anhydrous glucose powder within 5 minutes. The levels of blood glucose, insulin and glucagon were measured at 0, 30, 60, 120, and 180 minutes, respectively. The plasma glucagon levels measured at the above-mentioned time points were described as Gluca0min, Gluca30min, Gluca60min, Gluca120min, and Gluca180min, respectively.

### Assessment of islet α-cell function

Glucagon levels were measured at multiple points through the OGTT test as mentioned above, and the area under the curve, AUCgla, was calculated to estimate the overall glucagon level. AUCgla was calculated based on the following function: AUCgla = 15 × Gluca0min + 30 × (Gluca30min + Gluca180min) + 45 × Gluca60min + 60× Gluc120min. The function of pancreatic isled α cell was reflected by plasma glucagon level.

### Electromyography

PNCV, PNCA and PNCL were measured using the Danish Keypoint electromyography in Nantong First People's Hospital. The measurement was performed by medical professionals without any external factors such as muscle strain or strenuous exercise before the examination and in an environment with a room temperature between 24‒26°C. SPSS 23.0 software was used to calculate the Z scores of PNC, PNCV, PNCA and PNCL, which were then used to determine the overall function of PNC.

### Statistical analyses

All data were analyzed using SPSS 23.0 software. Categorical data are presented as frequency (percentage), and the Chi-Square test was used for data comparison between groups. Continuous data were first tested for normality, and normally distributed data are presented as mean ± Standard Deviation (SD). The independent *t*-test was used for data comparison between two groups, and one-way analysis of variance (ANOVA) was used for data comparison among multiple groups. Data with non-normal distribution are presented as the median (25% and 75%), and the non-parametric test was used for data comparison between groups. Spearman rank correlation and linear regression were used to analyze the correlations between Gluca30min and PNCV, PNCA and PNCL, respectively. Logistic regression was used to identify the potential risk factors of DPN, and the ROC curve was used to evaluate the predictive value of plasma glucagon levels for DPN. A p-value less than 0.05 was considered statistically significant.

## Results

### Comparison of demographic and baseline clinical characteristics between two groups

As shown in Table 1, the DPN group had an older age and longer disease duration than the NDPN group. Also, the DPN group had lower plasma concentrations of ALT, AST, and 2h-C peptide, but higher plasma concentrations of urea nitrogen and HDL than the NDPN group. There were no significant differences with regard to gender, BMI, blood pressure and other biochemical indexes such as total cholesterol and triglyceride between these two groups. In addition, the DPN group had significantly lower Gluca30min, Gluca60min, Gluca120min and AUCgla than the NDPN group (p < 0.05 or 0.001), indicating that pancreatic α cell function decreased in T2DM patients with DPN.

**Table 1:** Comparison of demographic and baseline clinical characteristics between the two groups.

Clinical features	NDPN (n = 220)	DPN (n = 138)	T/Z/X^2^	p
Age (years)	54 (45‒60)	57 (49.75‒64)	−2.527	0.012
Gender (Female)	91 (41.4)	44 (31.9)	3.244	0.072
Disease duration (years)	3 (0.5‒8)	7 (2‒14)	−4.710	<0.001
BMI (kg/㎡)	25.39 (23.10‒27.67)	24.91 (22.50‒27.78)	−1.407	0.159
SBP (mmHg)	135.00 (124.00‒147.00)	132.00 (117.00‒143.00)	−1.540	0.124
DBP (mmHg)	83.00 (76.00‒90.75)	80.00 (72.50‒90.00)	−1.674	0.094
Alanine aminotransferase (U/L)	23.00 (17.00‒37.00)	18.00 (13.00‒28.00)	−4.047	0.001
Aspartate aminotransferase (U/L)	18.00 (15.00‒25.75)	17.00 (13.00‒21.05)	−2.506	0.012
Urea nitrogen(mmoL/L)	5.34 (4.32‒6.39)	5.79 (4.72‒7.19)	−2.899	0.004
Creatinine (μmoL/L)	59.50 (50.03‒69.58)	61.40 (51.65‒75.45)	−1.692	0.091
Uric acid (μmoL/L)	312.70 (266.30‒383.30)	313.90 (236.75‒409.45)	−0.077	0.939
Triglycerides (mmoL/L)	1.95 (1.33‒3.23)	1.75 (1.09‒2.97)	−1.914	0.056
Total cholesterol (mmoL/L)	4.49 (3.80‒5.21)	4.48 (3.99‒5.16)	−0.384	0.701
Low density lipoprotein (mmoL/L)	2.68±0.84	2.67±0.75	0.140	0.889
High-density lipoprotein (mmoL/L)	1.04 (0.88‒1.19)	1.09 (0.96‒1.24)	−2.802	0.005
Fasting blood glucose (mmoL/L)	6.26 (4.89‒7.63)	6.34 (5.08‒8.48)	−1.769	0.077
Glycated hemoglobin A1c (%)	9.75 (8.14‒11.20)	9.76 (8.33‒11.73)	−0.422	0.673
0h-C peptide (ng/mL)	1.65 (1.09‒2.54)	1.48 (0.86‒2.33)	−1.962	0.050
2h-C peptide (ng/mL)	6.25 (4.58‒8.01)	4.09 (3.05‒5.17)	−6.923	<0.001
Gluca0min (pmoL/L)	12.36 (8.27‒18.24)	11.37 (7.45‒15.93)	−1.347	0.178
Gluca30min (pmoL/L)	18.30 (12.77‒14.36)	12.26 (9.10‒18.26)	−6.309	<0.001
Gluca60min (pmoL/L)	12.37(8.76‒17.33)	10.31 (6.83‒13.92)	−3.50	0.002
Gluca120min (pmoL/L)	9.48 (6.62‒13.29)	7.61 (5.38‒11.09)	−3.009	0.003
Gluca180min (pmoL/L)	8.57 (5.44‒12.13)	8.29 (5.14‒11.71)	−1.048	0.295
AUCgla	2138.48 (1484.06‒2883.15)	1196.32 (966.99‒2348.62)	−3.741	0.001

### Correlation between glucagon levels and PNC function in T2DM patients with DPN

The authors next examined the correlation between plasma glucagon levels and PNC functions using the functional indexes PNCV, PNCA and PNCL. In patients with T2DM, the glucagon levels, mainly Gluca30min, Gluca60min, and gluca120min, were significantly positively correlated with PNCV (Table 2) and PNCA (Table 4) but negatively correlated with PNCL (Table 3). Of these, Gluca30min had the strongest correlation with PNC function (p < 0.05).

**Table 2:** Positive correlation between glucagon levels and PNCV in patients with T2DM.

	Gluca0min		Gluca30min		Gluca60min		Gluca120min		Gluca180min		AUCgla	
	*r*	p	r	p	*r*	p	*r*	p	*r*	p	*r*	p
**Left median nerve MCV**	0.057	0.279	0.202	0.001	0.108	0.041	0.127	0.016	0.068	0.197	0.139	0.009
**Right median nerve MCV**	0.094	0.075	0.218	0.002	0.122	0.021	0.135	0.011	0.05	0.348	0.143	0.007
**Left ulnar nerve MCV**	0.069	0.191	0.179	0.003	0.081	0.125	0.09	0.09	0.021	0.687	0.105	0.047
**Right ulnar nerve MCV**	0.062	0.241	0.214	0.002	0.113	0.033	0.111	0.035	0.033	0.535	0.128	0.016
**Left tibial nerve MCV**	0.142	0.007	0.264	<0.001	0.182	0.001	0.152	0.004	0.096	0.07	0.196	0.002
**Right tibial nerve MCV**	0.103	0.052	0.24	<0.001	0.17	0.001	0.157	0.003	0.07	0.185	0.179	0.001
**Left common peroneal nerve MCV**	0.126	0.017	0.26	<0.001	0.179	0.001	0.155	0.003	0.058	0.276	0.186	<0.001
**Right common peroneal nerve MCV**	0.151	0.004	0.277	<0.001	0.203	<0.001	0.157	0.003	0.094	0.076	0.206	<0.001
**Left median nerve SCV**	0.114	0.032	0.209	0.002	0.177	0.001	0.157	0.003	0.102	0.055	0.176	0.001
**Right median nerve SCV**	0.088	0.098	0.186	0.001	0.157	0.003	0.139	0.009	0.08	0.131	0.155	0.003
**Left ulnar nerve SCV**	0.106	0.046	0.232	0.002	0.186	<0.001	0.178	0.001	0.083	0.119	0.191	0.002
**Right ulnar nerve SCV**	0.108	0.043	0.243	0.002	0.19	<0.001	0.161	0.002	0.054	0.311	0.19	0.003
**Left superficial peroneal nerve SCV**	0.116	0.034	0.24	0.001	0.159	0.004	0.157	0.004	0.088	0.108	0.185	0.001
**Right superficial peroneal nerve SCV**	0.093	0.092	0.204	<0.001	0.182	0.001	0.175	0.001	0.091	0.099	0.186	0.001
**Left sural nerve SCV**	0.114	0.036	0.28	<0.001	0.227	<0.001	0.204	<0.001	0.135	0.013	0.231	<0.001
**Right sural nerve SCV**	0.090	0.094	0.204	<0.001	0.201	<0.001	0.177	0.001	0.118	0.028	0.195	<0.001

**Table 3:** Positive correlation between glucagon levels and PVCN in patients with T2DM.

			Gluca0min		Gluca30min		Gluca60min		Gluca120min		Gluca180min		AUGgla	
			*r*	p	*r*	p	*r*	p	*r*	p	*r*	p	*r*	p
**Sports**	**Left median nerve latency**													
	Wrist-APB		0.005	0.924	0.079	0.136	0.017	0.745	−0.006	0.914	−0.053	0.322	0.018	0.728
	Elbow-wrist		−0.015	0.771	0.062	0.239	−0.006	0.907	−0.022	0.675	−0.059	0.269	0	0.999
	**Right median nerve latency**													
	Wrist-APB		0.0202	0.705	0.097	0.069	0.071	0.182	−0.003	0.962	−0.022	0.683	0.042	0.426
	Elbow-wrist		0.0120	0.824	0.070	0.187	0.033	0.533	−0.027	0.617	−0.038	0.476	0.015	0.782
	**Left ulnar nerve latency**													
	Wrist-ADM		0.058	0.271	0.100	0.059	0.051	0.331	−0.003	0.962	0.022	0.678	0.049	0.360
	Below elbow - wrist		0.043	0.415	0.089	0.094	0.041	0.444	−0.006	0.908	−0.002	0.962	0.034	0.524
	**Right ulnar nerve latency**													
	Wrist-ADM		0.007	0.893	0.102	0.055	0.032	0.550	−0.038	0.472	0.024	0.644	0.027	0.615
	Below elbow - wrist		−0.014	0.787	0.086	0.103	0.022	0.675	−0.035	0.509	0.018	0.735	0.018	0.732
	**Left tibial nerve latency**													
	Ankle-AH		−0.050	0.343	0.048	0.368	0.018	0.730	0.066	0.210	−0.041	0.435	0.032	0.540
	Popliteal fossa-ankle		−0.062	0.242	0.041	0.441	0.016	0.757	0.034	0.518	−0.057	0.278	0.016	0.769
	**Right tibial nerve latency**													
	Ankle-AH		0.013	0.799	0.101	0.057	0.070	0.188	0.088	0.095	−0.024	0.651	0.071	0.183
	Popliteal fossa-ankle		0.021	0.698	0.126	0.017	0.091	0.086	0.089	0.094	−0.02	0.710	0.085	0.107
	**Left common peroneal nerve latency**													
	Ankle-EDB		0.152	0.004	0.173	0.001	0.144	0.007	0.130	0.014	0.057	0.283	0.145	0.006
	below knee-ankle		0.116	0.028	0.176	0.001	0.152	0.004	0.121	0.023	0.042	0.425	0.141	0.008
	**Right common peroneal nerve latency**													
	Ankle-EDB		0.077	0.148	0.127	0.017	0.097	0.068	0.113	0.032	0.061	0.249	0.11	0.038
	below knee-ankle		0.090	0.090	0.169	0.001	0.129	0.015	0.141	0.008	0.073	0.171	0.141	0.008
**Sensation**	Left median nerve latency		−0.009	0.870	0.115	0.030	0.017	0.750	0.007	0.888	−0.060	0.256	0.031	0.565
	Right median nerve latency		0.032	0.550	0.167	0.002	0.080	0.132	0.063	0.237	−0.015	0.777	0.091	0.087
	Left ulnar nerve latency		−0.077	0.147	0.095	0.074	−0.006	0.907	0.007	0.888	−0.116	0.029	0.005	0.920
	Right ulnar nerve latency		0.026	0.630	0.142	0.008	0.056	0.290	0.025	0.640	−0.089	0.093	0.055	0.301
	Left superficial peroneal nerve latency		0.081	0.142	0.179	0.001	0.090	0.102	0.085	0.120	0.013	0.811	0.115	0.036
	Right superficial peroneal nerve latency		0.069	0.209	0.155	0.005	0.063	0.255	0.052	0.347	−0.024	0.666	0.079	0.153
	Left sural nerve latency		0.014	0.797	0.153	0.005	0.054	0.324	0.038	0.481	−0.043	0.433	0.061	0.260
	Right sural nerve latency		0.026	0.626	0.173	0.001	0.090	0.094	0.093	0.086	0.006	0.916	0.103	0.055

**Table 4:** Negative correlation between glucagon levels and PNCL in patients with T2DM.

			Gluca0min		Gluca30min		Gluca60min		Gluca120min		Gluca180min		AUGgla	
			*r*	p	*r*	p	*r*	p	*r*	p	*r*	p	*r*	p
**Sports**	**Left median nerve latency**													
		Wrist-APB	-0.094	0.074	-0.179	0.001	-0.136	0.010	-0.150	0.004	-0.066	0.210	-0.145	0.006
		Elbow-wrist	-0.073	0.166	-0.193	<0.001	-0.132	0.012	-0.176	0.001	-0.068	0.198	-0.155	0.003
	**Right median nerve latency**													
		Wrist-APB	-0.044	0.406	-0.164	0.009	-0.137	0.010	-0.136	0.010	-0.045	0.395	-0.131	0.014
		Elbow-wrist	-0.061	0.250	-0.195	<0.001	-0.149	0.005	-0.179	0.001	-0.069	0.191	-0.16	0.002
	**Left ulnar nerve latency**													
		Wrist-ADM	-0.141	0.008	-0.245	<0.001	-0.189	<0.001	-0.184	<0.001	-0.091	0.086	-0.2	<0.001
		Below elbow - wrist	-0.054	0.305	-0.168	0.001	-0.081	0.124	-0.108	0.040	-0.005	0.931	-0.104	0.049
	**Right ulnar nerve latency**													
		Wrist-ADM	-0.112	0.034	-0.211	<0.001	-0.127	0.016	-0.123	0.020	-0.072	0.176	-0.148	0.005
		Below elbow - wrist	-0.065	0.223	-0.178	0.001	-0.087	0.102	-0.113	0.033	-0.014	0.797	-0.111	0.035
	**Left tibial nerve latency**													
		Ankle-AH	-0.097	0.066	-0.151	0.004	-0.093	0.080	-0.102	0.053	-0.043	0.420	-0.111	0.037
		Popliteal fossa-ankle	-0.083	0.115	-0.176	0.001	-0.104	0.049	-0.116	0.028	-0.020	0.700	-0.122	0.020
	**Right tibial nerve latency**													
		Ankle-AH	-0.096	0.070	-0.14	0.008	-0.075	0.155	-0.109	0.039	-0.049	0.356	-0.107	0.043
		Popliteal fossa-ankle	-0.068	0.199	-0.159	0.003	-0.085	0.108	-0.114	0.031	-0.006	0.904	-0.109	0.040
	**Left common peroneal nerve latency**													
		Ankle-EDB	-0.089	0.095	-0.145	0.006	-0.106	0.045	-0.122	0.021	-0.025	0.634	-0.114	0.031
		below knee-ankle	-0.083	0.117	-0.111	0.036	-0.109	0.040	-0.112	0.034	-0.058	0.273	-0.103	0.052
	**Right common peroneal nerve latency**													
		Ankle-EDB	-0.079	0.137	-0.122	0.021	-0.088	0.096	-0.121	0.022	-0.062	0.246	-0.104	0.051
		Below knee-ankle	-0.079	0.136	-0.159	0.003	-0.093	0.080	-0.118	0.026	-0.035	0.511	-0.112	0.034
**Sensation**	Left median nerve latency		-0.06	0.258	-0.189	0.001	-0.143	0.007	-0.152	0.004	-0.051	0.334	-0.15	0.004
	Right median nerve latency		-0.032	0.554	-0.153	0.004	-0.103	0.052	-0.108	0.042	-0.015	0.779	-0.105	0.047
	Left ulnar nerve latency		-0.046	0.390	-0.164	0.002	-0.087	0.101	-0.113	0.033	0.018	0.732	-0.107	0.045
	Right ulnar nerve latency		-0.048	0.373	-0.197	<0.001	-0.108	0.042	-0.095	0.075	0.027	0.610	-0.114	0.032
	Left superficial peroneal nerve latency		-0.089	0.103	-0.183	0.001	-0.110	0.044	-0.143	0.009	-0.016	0.770	-0.142	0.009
	Right superficial peroneal nerve latency		-0.104	0.057	-0.219	0.001	-0.150	0.006	-0.158	0.004	-0.037	0.505	-0.168	0.002
	Left sural nerve latency		-0.084	0.122	-0.211	<0.001	-0.127	0.020	-0.139	0.011	-0.060	0.271	-0.152	0.005
	Right sural nerve latency		-0.078	0.149	-0.175	0.002	-0.096	0.074	-0.107	0.048	-0.003	0.960	-0.114	0.035

Next, the authors performed quartile grouping analysis based on the Gluca30min values (Q1, Q2, Q3 and Q4 groups), and calculated the Z scores of PNCV, PNCA and PNCL. The authors found that the Z scores of PNCV and PNCA each showed an increasing trend from Q1 to Q4 (p < 0.05), while the Z score of PNCL showed a decreasing trend (p < 0.05) (Table 5).

**Table 5:** Comparison of demographic and clinical characteristics of four quartile groups of T2DM patients.

Variables	Total number	Q1	Q2	Q3	Q4	*X*^2^/F	p
Gluca30min	15.80 (11.01‒22.35)	8.59 (6.78‒10.03)	13.16 (12.23‒13.16)	18.59 (17.31‒20.37)	26.28 (24.15‒32.20)	‒	‒
n	358	89	90	90	89	‒	‒
Age (years)	55 (47‒62.0)	58.5 (51.25‒65.0)	56 (47‒61)	54 (44‒60)	55 (43‒60)	11.374	0.010
Gender (Female)	135 (37.7)	36 (40.4)	36 (40.4)	30 (33.3)	33 (37.1)	1.234	0.745
DPN	138 (38.5)	57 (64.0)	35 (38.9)	34 (37.8)	12 (13.5)	48.056	0.001
Duration of disease (years)	5 (0.65‒10)	6.50 (1.00‒12.75)	6.00 (2.00‒10.00)	4.00 (0.88‒10.00)	2.00 (0.5‒8)	10.986	0.012
Body mass index (kg/m^2^)	25.2 (22.86‒27.69)	24.15 (21.91‒27.24)	24.94 (22.98‒26.81)	25.35 (23.11‒28.03	26.80 (23.46‒28.69)	14.041	0.003
Systolic blood pressure (mmHg)	133 (121‒145)	130 (117.25‒141)	134.5 (120‒145.50)	132 (120.75‒143.25)	140 (127.50‒152.00)	11.793	0.008
Diastolic blood pressure (mmHg)	82(75‒90)	80.00 (73.0‒87.75)	82.50 (72.75‒90.25)	82.00 (76.0‒91.00)	85.00 (77.50‒92.5)	6.895	0.075
Alanine aminotransferase (U/L)	21.00 (15.00‒32.00)	18.00 (12.00‒29.00)	21.50 (16.25‒36.50)	20.00 (15.00‒29.50)	25.00 (17.00‒37.00)	12.855	0.005
Aspartate aminotransferase (U/L)	18.00(14.00‒24.00)	18.00 (13.00‒23.00)	18.00 (15.00‒25.75)	16.00 (13.00‒21.00)	18.00 (15.00‒25.25)	7.699	0.053
Urea nitrogen (mmoL/L)	5.44 (4.45‒6.76)	5.60(4.47‒7.60)	5.71 (4.55‒6.80)	4.97 (4.22‒5.89)	5.60 (4.70‒6.52)	9.827	0.020
Creatinine (μmoL/L)	60.10 (50.58‒71.50)	60.95 (50.28‒74.28)	57.85 (49.10‒69.88)	59.40 (52.4‒69.15)	62.55 (53.7‒73.93)	2.457	0.483
Uric acid (μmoL/L)	313.35 (255.08‒390.03)	306.55 (229.58‒390.45)	297.45 (241.65‒392.53)	319.90 (274.15‒385.7)	319.1 (268.95‒398.38)	2.556	0.465
Triglycerides (mmoL/L)	1.88 (1.25‒3.15)	1.54 (0.98‒2.32)	1.68 (1.15‒3.19)	2.27 (1.40‒3.70)	2.12 (1.40‒3.87)	-1.914	0.056
Total cholesterol (mmoL/L)	4.49 (3.87‒5.21)	4.51 (3.96‒5.09)	4.33 (3.76‒5.20)	4.62 (3.94‒5.55)	4.53 (3.76‒5.17)	-0.384	0.701
Low density lipoprotein (mmoL/L)	2.68±0.81	2.66±0.65	2.63±0.77	2.77±0.94	2.64±0.84	0.61	0.609
High-density lipoprotein (mmoL/L)	1.06 (0.91‒1.22)	1.11 (0.94‒1.27)	1.04 (0.92‒1.21)	1.04 (0.89‒1.20)	1.05 (0.90‒1.21)	-2.802	0.005
Diabetes treatment plan							
Simple lifestyle (%)	13 (3.6)	2 (2.2)	5 (5.6)	2 (2.2)	4 (4.5)	2.14	0.544
Insulin therapy (%)	155 (43.3)	55 (61.8)	35 (38.9)	34 (37.8)	31 (34.8)	16.835	0.001
Insulin secretagogue (%)	36 (10.1)	11 (12.4)	7 (7.8)	11 (12.2)	7 (7.9)	1.978	0.577
Biguanide drugs (%)	186 (52.0)	40 (44.9)	44 (48.9)	54 (60.0)	48 (53.9)	4.565	0.207
Insulin sensitizer (%)	54 (15.1)	12 (13.5)	17 (18.9)	12 (13.3)	13 (14.6)	1.426	0.699
Fasting blood glucose (mmoL/L)	6.27 (5.00‒7.80)	5.75 (4.53‒7.43)	6.09 (4.87‒7.93)	7.04 (5.53‒8.16)	6.27 (5.21‒7.22)	-1.769	0.077
Glycated hemoglobin A1c (%)	9.76 (8.19‒11.33)	9.96 (8.53‒11.90)	9.70 (7.85‒11.5)	9.86 (8.28‒11.53)	9.46 (7.99‒10.73)	-0.422	0.673
CP2h (ng/mL)	5.26 (3.59‒7.44)	4.36 (3.07‒6.72)	5.25 (3.39‒7.63)	5.08 (3.58‒6.86)	6.36 (4.87‒8.91)	28.431	<0.001
AUCgla	1972.2 (1359.64‒2688.22)	1195.45 (908.4‒1343.55)	1669.42 (1420.46‒1906.95)	2250.22 (2022.94‒2639.7)	3171.75 (2720.92‒3972.82)	-3.741	0.002
Composite Z-score for nerve conduction velocity	‒	‒0.46±1.12	0.03±0.99	0.02±0.97	0.41±0.69	12.269	<0.001
Composite Z-score for nerve conduction latency	‒	0.49±1.17	‒0.15±0.96	‒0.07±0.90	‒0.27±0.76	11.113	<0.001
Composite Z-score for nerve conduction amplitude	‒	‒0.39±1.05	0.13±0.97	‒0.06±0.96	0.32±0.88	8.551	<0.001

**Fig. 1:**
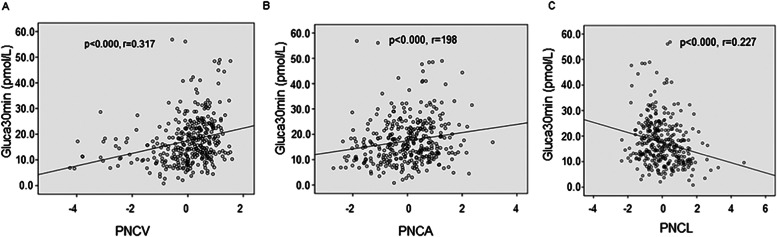
Gluca30min is positively correlated with PNCV (*r* = 0.137, A) and PNCA (*r* = 0.198, B) but negatively correlated with PNCL (*r* = -0.227, C).

Consistent with the above observations, linear correlation analysis showed that Gluca30min was positively correlated with PNCV (*r* = 0.317) and PNCA (*r* = 0.198) and negatively correlated with PNCL (*r* = -0.227) (all p < 0.05) (Fig. 1 A, B & C).

### Determination of risk factors for PNC function

The authors further used Gluca30min as the dependent variable in a multiple linear stepwise regression analysis to correct other clinical confounding factors and attempted to uncover a correlation with PNC function. The authors found that Gluca30min was independently correlated to PNCV, PNCA and PNCL (p < 0.05) (Table 6).

**Table 6:** Multiple linear regression analysis of correlation between Gluca30min and PNC function.

Models	β	t	p	R^2^ after correction
Comprehensive Z-score for PNCV				
Model 0	0.270	5.299	<0.001	0.071
Model 1	0.194	3.391	0.001	0.134
Comprehensive Z-score for PNCL				
Model 0	-0.218	-4.21	<0.001	0.045
Model 1	-0.166	-2.901	0.004	0.126
Comprehensive Z-score for PNCA				
Model 0	0.178	3.409	0.001	0.029
Model 1	0.103	1.715	0.048	0.112

Model 0: Uncorrected.

Model 1: After correction of age, disease duration, body mass index, systolic blood pressure, alanine aminotransferase, urea nitrogen, high-density lipoprotein, insulin treatment, 2h-C peptide (correction factors were included based on p<0.05 in Table 5).

The authors then performed logistic regression analysis to explore the correlation of Gluca30min with DPN. After incorporating the factors with p < 0.05 shown in Table 5, Gluca30min was independently correlated to DPN (p < 0.05; Table 7).

**Table 7:** Logistic regression analysis of correlation between Gluca30min and DPN.

Model	Regression coefficient	Standard error	Wald	p-value	Odds ratio (OR)	95% CI
Model 0	-0.091	0.017	30.401	<0.001	0.913	0.884‒0.943
Model 1	-0.089	0.017	27.275	<0.001	0.915	0.884‒0.946
Model 2	-0.086	0.018	23.663	0.002	0.917	0.886‒0.950
Model 3	-0.101	0.022	21.653	0.008	0.904	0.867‒0.943

Model 0: Uncorrected.

Model 1: After correction of age, body mass index, systolic blood pressure.

Model 2: Further correction of diabetes duration and insulin treatment.

Model 3: Alanine aminotransferase, urea nitrogen, high-density lipoprotein, and 2h-C peptide were further corrected (correction factors were included based on those with p < 0.05 in Table 5).

The authors next performed the ROC curve analysis to examine whether the identified factors had any predictive values for DPN. The authors found that AUCs of 2h-CP, disease duration, and Gluca30min in predicting DPN were 0.717 (p < 0.001), 0.646 (p < 0.001), and 0.698 (p < 0.001), respectively (Table 8). The sensitivity and specificity of these three factors for predicting the occurrence of DPN were 74.64% and 68.18% for 2h-CP, 55.07% and 69.09% for disease duration, and 82.61% and 48.18% for Gluca30min, respectively. Thus, Gluca30min can be used as an independent predictor for the occurrence of DPN as other two factors, 2h-CP and disease duration.

**Table 8:** ROC curve analysis of independent risk factors for predicting DPN.

Indicator	Threshold	Sensitivity (%)	Specificity (%)	AUC (95% CI)	p
2h-CP (ng/mL)	5.05	74.64	68.18	0.717 (0.668‒0.763)	<0.001
Disease duration	6	55.07	69.09	0.646 (0.594‒0.696)	<0.001
Gluca30min	18.68	82.61	48.18	0.698 (0.648‒0.745)	<0.001

## Discussion

T2DM is the most common DM and its prevalence has been increasing worldwide.^16^ DPN is the most frequent neurological complication in T2DM patients and significantly contributes to severe morbidity and mortality and economic burden for society.^17^ The present study investigated the correlation between the level of blood glucagon, which reflects the function of pancreatic α islet cells, and the PNC function, which is reflected by PNCV, PNCA, and PNCL. The authors found significant correlations between the plasma glucagon levels and the PNC function and between Gluca30min and DPN in T2DM patients. The present findings support a significant link between the pancreatic α islet cell function and PNC function as well as DPN in T2DM patients.

It is well known that T2DM patients had higher plasma glucagon levels than the control subjects.^18^^,^^19^ However, few studies were performed to compare the plasma levels of glucagon in T2DM patients with and without DPN. Therefore, it is unclear whether there is any correlation between plasma glucagon level and PNC function and DPN. In the present study, the authors found that the NDPN group had higher glucagon levels at 30, 60 and 120 min and AUCglu after sugar loading than the DPN group. DPN has an insidious onset in the early stage, slow progression, and complex pathogenesis. One previous study showed that mice deficient in Glucagon Gene-Derived Peptides (GCGDPs) are prone to peripheral neuropathy as they age.^20^ Supplementation with physiological concentrations of glucagon (10^−7^ and 10^−6^ moL/L) did not have any significant effects on the neurites of mice, however, glucagon promotes the growth of mouse neurites at a supraphysiological concentration (10^−5^ moL/L).^20^ Another study suggested that glucagon can reduce the toxicity of Methylglyoxal (MG) to mouse ganglion neuron cells, increase cell survival rate, and accelerate neurite growth.^21^ Thus, plasma glucagon appears to be beneficial to peripheral nerve function, which can be assessed by measurements of PNCV, PNCA and PNCL.^22^ The authors observed that the increases in glucagon levels, particularly Gluca30min, and AUCgla, were accompanied by increased PNCV and PNCA but decreased PNCL. The activation of the autonomic nervous system was reported to promote glucagon secretion.^23^ When peripheral nerves are damaged, glucagon secretion is stimulated, thereby increasing the survival rate of neuronal cells and accelerating the growth of neuronal cells.^23^ Therefore, it can be speculated that elevated glucagon levels are beneficial to peripheral nerve function and can delay the occurrence or slow down the progression of DPN in T2DM patients to a certain degree. This speculation merits further interrogation because few studies were designed to postulate its beneficial effects on DPN. On the other hand, it is noteworthy that the exact mechanisms driving the development of DPN remain unclear, however, it is speculated that it is multifactorial, involving activation of the polyol pathway, an increase of Advanced Glycation end products (AGEs) and their receptors, activation of Protein Kinase C (PKC).^3^ In addition, insulin resistance, hyperlipidemia, increased oxidative stress and inflammatory response all contribute to the pathogenesis of DPN.^3^ Therefore, given the complex mechanisms underlying the DPN pathogenesis, whether and how increased plasma glucagon levels interact with other factors to play a role in the pathogenesis of DPN in T2DM patients warrants further investigation.

Normal human pancreatic islet α cells are located on the periphery of the islet, while β-cells are located in the center of the islet. Therefore, when the islet microcirculation is disrupted, the secretory function of β-cells located in the center of the islet is more severely impaired. In this study, the authors observed that 2h-CP levels, a well-recognized indicator of insulin secretion and pancreatic β-cell function,^24^ were significantly lower in the DPN group than in the NDPN group, consistent with a previous report.^25^ One community-based study on T2DM showed a close association between 2h-CP levels and the development of DPN,^26^ and other studies suggested a significant correlation between the duration of diabetes and DPN in T2DM patients.^27^^,^^28^ In agreement with the above reports, this study also suggested that 2h-CP and disease duration were independent predictors for the pathogenesis of DPN. In the present study, among all glucagon levels measured at different time points, the glucagon level at 30 minutes, Gluca30min, had the most significant association with the PNC function. In the quartile study for glucagon levels, and taking the Q1 group as a reference, the increase in Gluca30min was accompanied by the increased PNC function, with the Q4 group being significantly higher than the Q1 group. After correcting all other confounding factors such as age, disease duration, BMI, systolic blood pressure, alanine aminotransferase, urea nitrogen, high-density lipoprotein, insulin treatment, and 2h-C peptide, Gluca30min was still significantly correlated with PNCV, PNCA and PNCL. Logistic regression analysis further revealed that Gluca30min level was independently related to DPN. The ROC curve analysis revealed that the AUC for the 2h-C peptide was 0.717, for disease duration was 0.646, and for the Gluca30min was 0.698. Taken together, the present study suggested that plasma glucagon level is one of the factors that have a significant correlation with PNC function. The authors then reckon that Gluca30min can be a potentially valuable independent predictor for the occurrence of DPN in addition to 2h-C peptide and disease duration. Future large cohort prospective studies are warranted to further corroborate this conclusion.

Some limitations of this study should be noted. For example, this study was a retrospective study and exhibited intrinsic limitations such as sampling bias and no revelation of a cause-effect relationship. Also, this study had a limited sample size. Due to this sample size, the authors did not stratify the plasma glucagon levels for association with different severity of DPN. Thus, although the conclusion, that Gluca30min can be used as an independent predictor for the occurrence of DPN, is promising, it needs to be further corroborated in large cohort prospective studies in the future.

In conclusion, the authors report here that the increase in plasma glucagon levels in T2DM patients is closely correlated with PNCV, PNCA and PNCL, which reflect the peripheral nerve function. Given that the plasma glucagon levels reflect the function of pancreatic α islet cells, the authors believe that the function of pancreatic α islet cells is correlated with DPN. Considering that Gluca30min has the strongest correlation with DPN, the present findings suggest that Gluca30min could be potentially a valuable predictor for the development of DPN in T2DM patients.

## Ethical statement

The study design was reviewed and approved by the Human Study Review Committee of Affiliated Hospital 2 of Nantong University. The conduction of the study adhered to the Declaration of Helsinki involving research of human subjects, and all patients signed an informed consent when admitted to the study.

## Authors’ contributions

XW contributed to the conception and design of the study. YC contributed to the data collection and assembly. XW and YC contributed to the data analysis and interpretation. XW contributed to the initial drafting of the manuscript. XW and YC revised the manuscript. All authors have read and approved the final manuscript.

## Declaration of competing interest

The authors declare no conflicts of interest.
